# New Philippine species of *Spilosmylus* Kolbe (Neuroptera, Osmylidae)

**DOI:** 10.3897/zookeys.712.19883

**Published:** 2017-10-26

**Authors:** Davide Badano, Shaun L. Winterton

**Affiliations:** 1 Dipartimento di Scienze della Terra, dell’Ambiente e della Vita, Università degli Studi di Genova, Genova, Italy; 2 California State Collection of Arthropods, Sacramento, California, United States of America

**Keywords:** Osmylidae, Spilosmylinae, lance lacewings, Oriental region, Malesia, taxonomy

## Abstract

New species of lance lacewings, *Spilosmylus spilopteryx* **sp. n.** and *Spilosmylus tephrodestigma* **sp. n.**, are described from the Philippines and compared with congeners. Both species are characterised by a distinctive wing pattern, which in the case of *Spilosmylus spilopteryx* **sp. n.** is relatively spectacular among lacewings. An identification key to the species of *Spilosmylus* Kolbe known from the Philippines is also provided.

## Introduction

Osmylidae, or lance lacewings, are a small (ca. 225 extant species) family of Neuroptera whose oldest fossil crown group members are known from various Jurassic to Tertiary deposits (Khramov 2014a, b; Oswald 2017; Winterton et al. 2017), and with stem group fossil species known from the Late Permian and throughout the Triassic (Khramov 2014a; Makarkin et al. 2014). Osmylids are present in most biogeographical regions but are notably absent from the Nearctic region, although tertiary-aged fossils are described from the Eocene of the Green River Formation (Makarkin 2017). They are medium- to large sized neuropterans, usually characterized by strongly patterned wings, sometimes of unusual shape. Their larvae are unmistakable due to their elongate, lance-like mandibles and paired hooked apparatus (i.e., pseudopods) at the apex of the abdomen (Matsuno and Yoshitomi 2016). The larval biology remains one of the most obscure aspects of this lacewing family, since it remains very poorly known with a few exceptions (Brauer 1851; Hölzel and Weissmair 2002; Matsuno and Yoshitomi 2016; Winterton et al. 2017). Osmylidae are also unusual among Neuroptera, with larvae in both riparian (e.g., wet soil adjacent to lotic water bodies) and terrestrial (e.g. leaf litter and subcortical) habitats (New 2003; Winterton et al. 2017). The phylogenetic position and internal relationships of Osmylidae have been long disputed, despite most authors agreeing that Osmylidae represent a relatively early branching lineage of Neuroptera (Withycombe 1925; Haring and Aspöck 2004; Aspöck and Aspöck 2008; Beutel et al. 2010; Randolf et al. 2014; Winterton et al. 2010). This phylogenetic position has been recently confirmed by mitogenomic studies, which recovered the family near the base of lacewing tree, more derived than Coniopterygidae and as a subsequent clade including Sisyridae + Nevrorthidae, sister to all the remaining families of Neuroptera (Wang et al. 2016). In their study of Osmylidae phylogeny, Winterton et al. (2017) found evidence of a sister relationship with Nevrorthidae and support for eight monophyletic osmylid subfamilies. These subfamilies were grouped into two main clades, the first including Gumillinae, Protosmylinae, Spilosmylinae and the second including Osmylinae, Porisminae, Eidoporisminae, Kempyninae and Stenosmylinae. The monophyly of the first lineage is supported by the unbranched hind wing vein CuP (in contrast with a strongly pectinate hind wing CuP vein of other subfamilies). A close relationship between Protosmylinae and Spilosmylinae is supported by molecular data and by the presence of unique features in male and female genitalia, in particular the presence of a narrowly arching gonarcus (Winterton and Wang 2016; Winterton et al. 2017). Spilosmylinae are recognizable due to the presence in the hind wing of a spur vein originating basal to the MP vein and of a basal sclerotised process on the mediuncus (Wang et al. 2011; Winterton et al. 2017). This group is by far the largest subfamily of osmylids, with at least 113 described species, although placed in only three genera: *Thaumatosmylus* Krüger (8 species), *Thyridosmylus* Krüger (20 species) and the most diverse genus of the family, *Spilosmylus* Kolbe (85 species). *Thaumatosmylus* is limited to the Oriental region (New 1991; Wang et al. 2011), while *Thyridosmylus* and *Spilosmylus* have a wider distribution, being also present in the Afrotropical and (in the case of *Spilosmylus*) Australasian regions, although they are most diverse in South-East Asia (Tjeder 1957, New 1986a, 1986b, 1988, 1991, 2003; Winterton et al. 2017). Divergence time estimates support a mid-Jurassic origin for *Thyridosmylus* and *Spilosmylus*, also explaining their unusual biogeographic pattern (Wang et al. 2011; Winterton et al 2017). Despite their ancient origin, the genera of Spilosmylinae are notoriously difficult to delimit using morphology alone and some species are of problematic allocation (New 2003). In particular, *Spilosmylus* is morphologically diverse, including both small and delicate (often yellow-green) species to large robust ones (New 2003). The genus *Thyridosmylus* and most species of *Spilosmylus* are best distinguished from *Thaumatosmylus* in the absence of crossveins between M and CuA after the basal crossvein, making a long undivided cell (New 1991, 2003). *Thyridosmylus* itself is mostly recognizable due to the presence of fenestrate markings on the forewing, although this distinction is unclear in some species as they lack the markings. Most species of *Spilosmylus* have intermittent dark dash-like markings between forewing veins Sc and R and/or the presence of an embossed spot (rarely two spots) near the hind margin of the forewing, although these characters are also highly variable (New 1986a, 2003) and are lacking in multiple species. The biology of Spilosmylinae is poorly known. The larvae of a Japanese species were reportedly found near streams (Kawashima 1957).

Malesia is a centre for diversification for *Spilosmylus*, with at least 54 species known from this region (New 2003). Various authors have described species from Malesia (McLachlan 1870; Gerstaecker 1893; Krüger 1913, 1914, Navás 1926), although their descriptions are often inadequate to provide useful comparisons. Banks (1924, 1931, 1937) described several species of *Spilosmylus*, particularly focussing on the Philippines and published the first identification key for species known from this archipelago (Banks 1937). Later, New (1986a, 1986b, 1988, 1991) revised the Oriental and Australasian Osmylidae, describing many new species of Spilosmylinae and provided identification keys to most of the known species. The works of New represent a significant contribution to the characterization of problematic and poorly known species described by earlier authors, and documents the exceptional diversity of *Spilosmylus* in the region. Despite these efforts, the lance lacewings of the Philippines remain poorly known and they received no further attention since then.

Herein, we describe two new species of *Spilosmylus, S. spilopteryx* sp. n. and *S. tephrodestigma* sp. n., from Luzon and compare them with the other species of *Spilosmylus* known from the Philippines. Both species are easily recognizable due to the distinctive wing pattern, easily setting apart them from all other congeners.

## Materials and methods

During the last few decades, two different terminology systems were applied to the genital sclerites of Osmylidae. Wang et al. (2011), Winterton and Wang (2016) and Winterton et al. (2017) used an updated version of the classical terminology of Tjeder (1957) and Adams (1969), based on comparisons and homology assessments across the whole family and with other Neuroptera. On the other hand, Aspöck and Aspöck (2008) proposed a different terminology, which was recently extensively applied to Osmylidae by Martins et al. (2016). However, as discussed in length by Winterton et al. (2017), the lack of adequate comparisons among the numerous subfamilies of Osmylidae hampered the recognition of genital sclerites in this family. In some subfamilies of Osmylidae the parameres (*sensu* Tjeder 1957) are absent, and the mediuncus has the role of main intromittent organ (Winterton et al. 2017). Therefore, Aspöck and Aspöck (2008) and Martins et al. (2016) considered the dorso-caudal sclerite as homologous with the gonocoxites 9 (i.e., parameres of Tjeder 1957), and the gonocoxites 11 (i.e., gonarcus of Tjeder 1957) as absent. However, in Protosmylinae, Spilosmylinae and Osmylinae the parameres are indeed present, and the gonarcus has an inverted “U”-shape typical of many neuropterans (Wang and Winterton 2016) (Fig. 4). Consequently, Osmylidae are in fact no exception with respect to other lacewing families in the overall structure of the genitalic sclerites, although the parameres have been lost in some subfamilies (e.g., Stenosmylinae, Kempyninae, Porisminae). To promote an interchangeability between the two terminologies used in Neuroptera, we consider the gonocoxites 11 *sensu* Aspöck and Aspöck (2008) as present in Osmylidae and homologous with the gonarcus of Tjeder (1957). Here we follow the terminology of Tjeder (1957) as implemented by Winterton et al. (2017).

Wing terminology follows Winterton et al. (2017) and does not assume that MA is fused basally with R to thus represent the posterior most vein of the R field.

Specimens were studied with a Leica MZ 9.5 stereomicroscope and measured with an optical micrometre. Photographs were taken with a Canon EOS 600D digital camera equipped with Canon lens MP-E 65 mm. The obtained images were stacked with the software Zerene Stacker and later post-processed with Adobe Photoshop. Specimens were measured using the following protocol: body length was taken from vertex to tip of the abdomen; wing length was measured longitudinally from base to apex, and wing width was taken as the maximum width perpendicular to the length measurement line. Genitalia were macerated in 10% KOH (potassium hydroxide) at room temperature, later rinsed in acetic acid and water and finally stained in Chlorazol Black. The genitalia were preserved in glycerol in a small vial put beneath the specimen.

## Taxonomy

### Spilosmylus spilopteryx sp. n.

Taxon classificationAnimaliaNeuropteraOsmylidae

http://zoobank.org/4FCE4CE0-E7E1-4A4A-A80E-C822F20513CF

**Figure 1.**
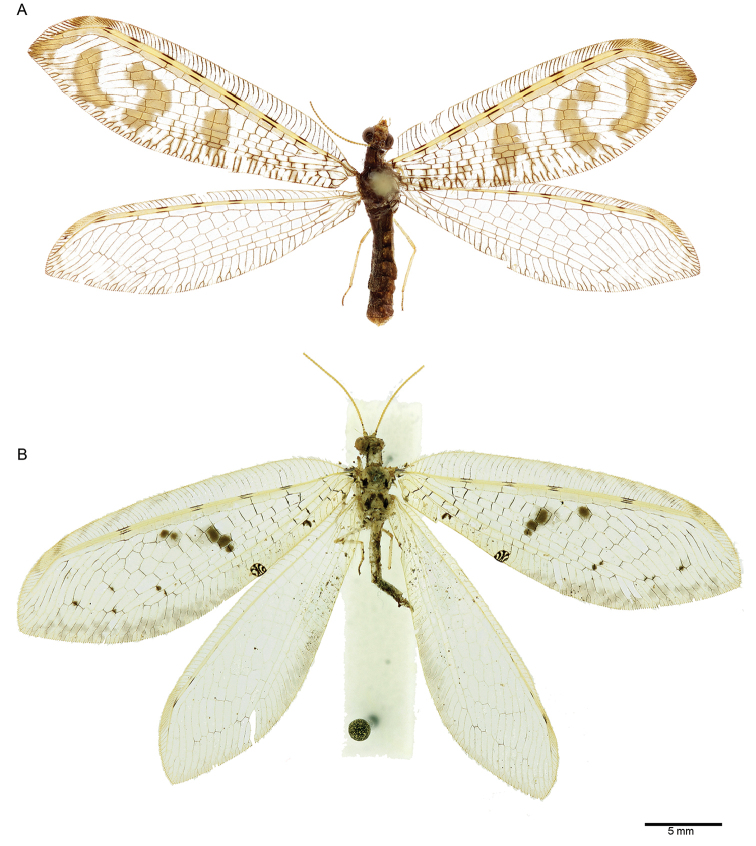
*Spilosmylus* spp., habitus: **A** *Spilosmylus spilopteryx* sp. n. **B** *Spilosmylus tephrodestigma* sp. n.

**Figure 2.**
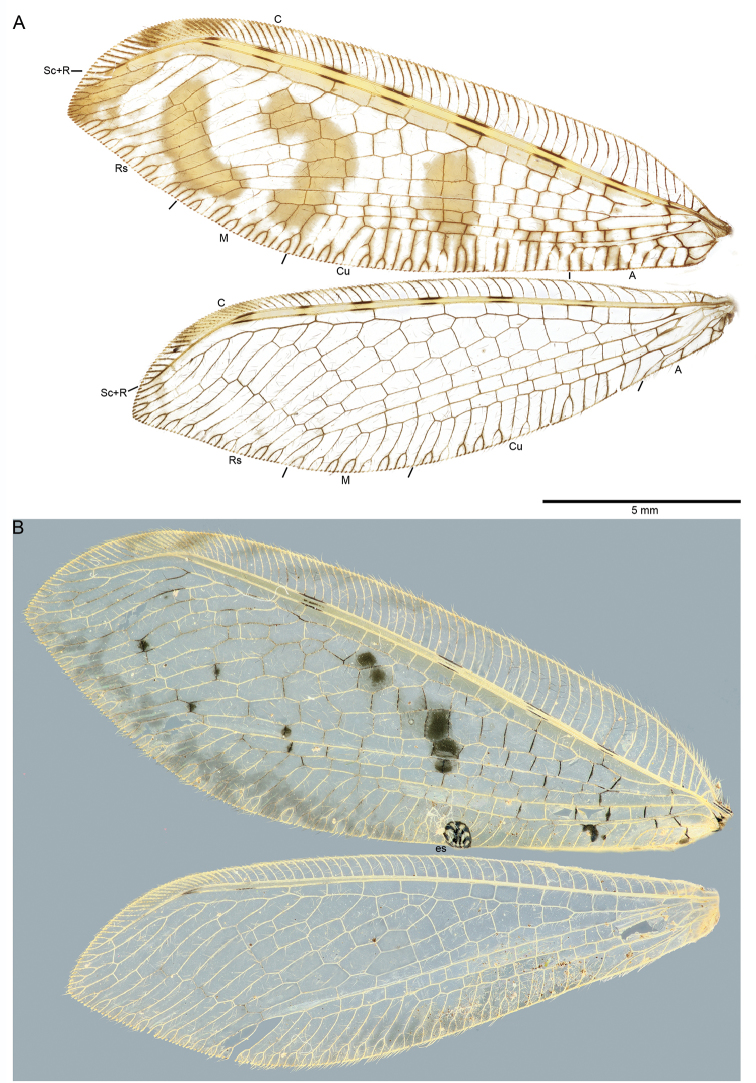
Wings of *Spilosmylus* spp. with main fields labeled: **A** *Spilosmylus spilopteryx* sp. n. **B** *Spilosmylus tephrodestigma* sp. n. Abbreviations: C, Costa; Sc, Subcosta; R, radius; Rs, Radius sector; M, Media; Cu, Cubitus; A, Anal field; es, embossed spot.

**Figure 3.**
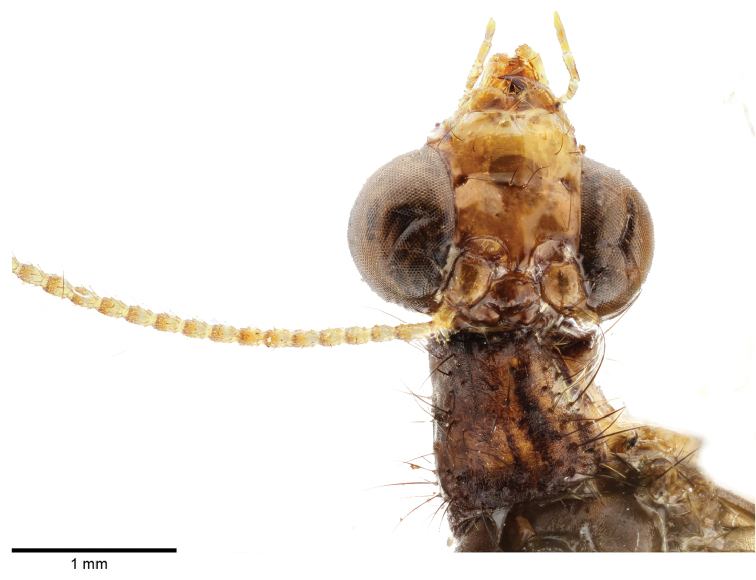
*Spilosmylus spilopteryx* sp. n. detail of the head and prothorax.

**Figure 4.**
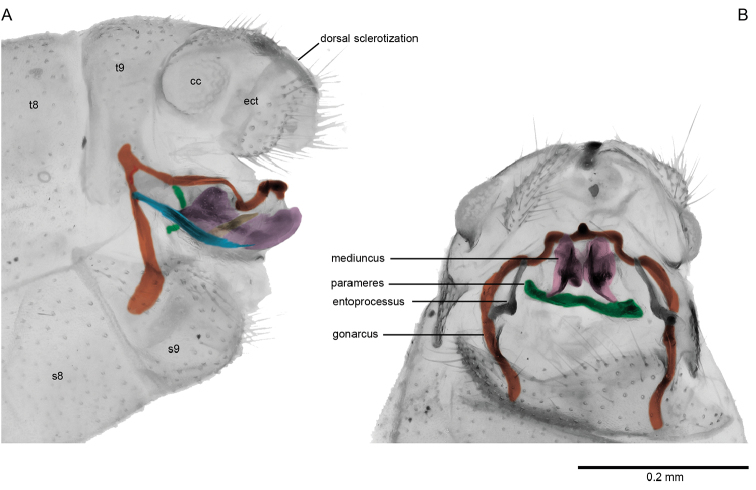
Male genitalia of *Spilosmylus spilopteryx* sp. n.: **A** lateral view **B** ventral view. Abbreviations: t8, tergite 8; s8, sternite 8; t9, tergite 9; st9, sternite 9; ect, ectoproct; cc, callus cercus.

Figs 1A , 2A , 3 , 4

#### Material examined.

**Holotype.** Pinned, genitalia in glycerol, preserved beneath the specimen. **PHILIPPINES**, South Luzon, Tigaon, Camerines sur, February 2015, 1 ♂, local collector, (Naturhistorisches Museum Wien).

#### Diagnosis.

Medium sized osmylid with uniformly brown body; both wings with intermittent dark dashes on Sc and R; forewing membrane with a distinct pattern composed by three large light brown markings; hind wing membrane hyaline (Fig. 1A).

#### Description.

*Dimensions.* Body length: 10.48 mm; forewing length 17.46 mm, width 6.03 mm; hind wing length: 16.35 mm, width: 5.08 mm.

*Head.* Mostly brown. Vertex light brown. Frons and clypeus reddish brown with a central rounded darker marking. Labrum and gena light reddish brown. Maxillary and labial palpi pale. Scape reddish brown, flagellomeres yellowish, slightly darker apically.

*Thorax*. Predominantly brown. Pronotum distinctly longer than wide, with undefined paler stripes running longitudinally to it (Fig. 3); mesonotum and metanotum uniformly brown; thorax covered with dark setae. Legs. Pale brown.

*Wings*. Forewing relatively broad with a slightly pointed apex, membrane hyaline with conspicuous markings and shades (Fig. 2A). Venation brown. Costal area progressively narrowing toward the apex. Pterostigma brown, lighter medially. Sc and R yellowish, with intermittent, parallel, black dashes. Subcostal area uniformly yellowish. Area between R and Rs uniformly light brown from the first crossvein until the pterostigmal area. Apex of forewing with a brown marking. Forewing medial fork clearly basal to the first branch of Rs. Forewing membrane with a diagnostic pattern composed by: a basal large, oval brown marking present at forewing middle length, a median elongated marking curved outward in proximity of the internal gradates and an apical elongated marking curved inward covering the external gradates (Fig. 2A). The latter marking is slightly more contrasted than the other two marks. MP, CuA, CuP and anal veins shaded with dark brown and with darkened crossveins. The apex of most veins reaching the hind margin is darkened. Embossed spot absent. Hind wing relatively broad. Sc and R yellowish, with intermittent, parallel, black dashes. Subcostal area yellowish like in the forewing but the rest of the membrane is unmarked with the exception of a few slightly shaded veins along the hind margin (Fig. 2A).

*Abdomen.* Tergites and sternites uniformly brown. Apex of the abdomen slightly lighter.

*Male genitalia.* Tergite 9 relatively narrow, extending slightly beyond the ectoproct. Sternite 9 subrectangular. Ectoproct rounded, with a prominent and relatively large callus cercus. Between the two halves of the ectoproct there is a narrow dorsal sclerotization curved downward (Fig. 4A, B). Parameres fused dorsally in an arch-shaped sclerite, rod-like in lateral view (Fig. 4A, B). Mediuncus relatively large, characterized by conspicuous distal paired flanges, connected to the gonarcus by membranes (Fig. 4A, B). Gonarcus narrow, arch-shaped, extending ventro-proximally as a flattened rod; distal section of the gonarcus in lateral view distinctly curved downward then bending up again in an almost straight apex; apical section of the gonarcus with a strongly sclerotized median thickening (Fig. 4A, B). Gonarcus equipped with a posterior entoprocessus extending posteriorly, bordering the mediuncus and narrowing apically (Fig. 4A, B).

**Etymology.** The specific name is a Latinized composite noun of Greek derivation, from *σπίλος*, *spilos*, meaning “marking” and the noun *πτέρυξ*, *pteryx*, meaning “wing”, thus *spilopteryx*, “marked wing”, in reference to the large cloud-like markings on the forewing.

#### Comments.

**Figure 5.**
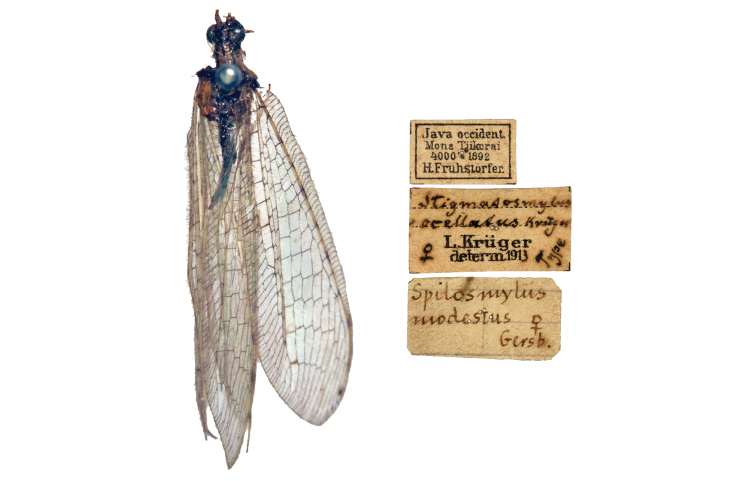
*Spilosmylus ocellatus* (Krüger, 1914), holotype, habitus and labels (Natural History Museum, Vienna).

*Spilosmylus spilopteryx* sp. n. is a highly distinctive species that cannot be easily confused with any other lance lacewing. This new species of *Spilosmylus* is characterized by a strongly marked wing and the absence of embossed spot on the hind margin of forewing, resembling the condition observed in the closely related genus *Thyridosmylus*. Nevertheless, overall wing shape and venation, the intermittently dashed markings along the Sc–R space, and male genitalic morphology allows us to confidently allocate this species to *Spilosmylus*. The presence of a narrow, dorsal sclerotization between the two halves of the ectoproct is characteristic of many species of *Spilosmylus*, and it might be of systematic relevance within this large genus. Despite several *Spilosmylus* species being characterized by pigmented wings with markings, bands and suffusions (e.g., *S. monticolus* (Banks, 1937), *S. formosus* Banks, 1924, *S. inquinatus* (McLachlan, 1870)), none of them display the extensive and conspicuous markings of this new taxon. Following New (1986, 1991), *Spilosmylus spilopteryx* sp. n. appears similar to *S. ocellatus* (Krüger, 1914) but strongly differing in the shape, extent and contrast of forewing markings. In particular, New (1986a) considered *S. ocellatus* as an easily recognizable species thanks to its wing pattern, which vaguely resembles the new species in his drawings, although composed by lighter shading and poorly contrasted markings (New 1986a: figs 115–116, New 1991). Nevertheless, the type specimen of *S. ocellatus*, preserved in the Naturhistoriches Museum Wien (Austria) bears no trace of such intense shading and its wing membrane appears mostly hyaline (Fig. 5). Noteworthy, a hand label of Navás suggest that the latter author also mistook this specimen for the inconspicuously marked *S. modestus* (Gerstaecker, 1893) (as also noted by Krüger 1914) (Fig. 5). Based on the examination of the type material of Krüger, we consider *S. ocellatus* and *S. spilopteryx* sp. n. as two very different taxa only sharing the lack of embossed spot. Further specimens are necessary to assess the identity of the morphospecies attributed by New (1986a) to *S. ocellatus*.

### Spilosmylus tephrodestigma sp. n.

Taxon classificationAnimaliaNeuropteraOsmylidae

http://zoobank.org/7F6F7D1B-38FA-42F3-8215-06BD9F174523

Figs 1B , 2B

#### Material examined.

**Holotype.** Pinned, abdomen damaged by booklice, genitalia missing. **PHILIPPINES**, North Luzon, Barlig, Mountain Province, July 2014, 1 ex, local collector, [gender indeterminate] (Naturhistorisches Museum Wien).

#### Diagnosis.

Medium sized osmylid with pale body; meso- and metathorax with large brown markings; both wings with small intermittent dark dashes on Sc and R; forewing membrane with well contrasted dark grey spots in the radial and medial area; base of the anal area with a well distinct dark marking; embossed spot present; hind wing membrane hyaline (Fig. 1B).

#### Description.

**Dimensions.** Forewing length: 21.43 mm, width: 7.14; hind wing length: 19.05 mm, width: 5.87 mm.

*Head.* Uniformly pale ochre. Vertex, frons and clypeus pale. Labrum, gena and palpi pale. Antenna uniformly pale ochre (Fig. 1B).

*Thorax*. Predominantly pale ochre. Pronotum distinctly longer than wide, with brown lateral margins; mesonotum with dark brown dots on the posterior portion of the mesoscutum; metanotum with dark brown markings on the metascutum converging apically on the prescutum (Fig. 1B). Legs. Pale.

*Wings*. Forewing relatively broad with a slightly pointed apex, membrane hyaline with isolated markings and shades (Fig. 2B). Venation of the costal area mostly pale, longitudinal veins predominantly yellowish, crossveins mostly brown. Costal area progressively narrowing toward the apex, with brownish shades toward the pterostigma. Pterostigma light brown, lighter medially. Sc and R yellowish, with 4 parallel black dashes. Subcostal area yellowish with dark streaks paralleling the dark dashes on Sc and R. Forewing medial fork originating basally to the first branch of Rs. Forewing membrane with a diagnostic pattern composed by: a dark grey marking between the origin of the third and fourth branches of Rs, and a series of three dark grey markings forming a stripe extending between the second branch of Rs and MP (Fig. 2B). Gradates with isolated dark spots. Basal cubital and anal crossveins blackish. Anal area with a characteristic curved dark marking at middle length between the wing base and the embossed spot (Fig. 2B). Posterior margin of the wing shaded. Hind wing relatively broad, with hyaline membrane. Venation predominantly yellowish. Posterior margin shaded.

*Abdomen.* Tergites and sternites uniformly pale ochre. Tip of the abdomen not preserved.

#### Etymology.

The specific epithet is a compound Latinized noun of Greek derivation from *τεφρῶδες*, *tephrodes*, meaning “coal” and *στίγμα*, *stigma*, meaning “spot”, thus “ashy spot” referring to the grey spots on the forewing.

#### Comments.

*Spilosmylus tephrodestigma* sp. n. is a more typical species of *Spilosmylus*, displaying a conspicuous embossed spot on the posterior margin of the forewing, which is an autapomorphic character of many species in the genus (Wang et al. 2011). *Spilosmylus tephrodestigma* sp. n. is also easily recognizable from other congeners thanks to the highly characteristic wing pattern composed by a series of dark grey spots forming a linear pattern in the radial area of forewing. *Spilosmylus tephrodestigma* sp. n. is similar to *S. inquinatus* and it might be closely related to the latter, but it is easily set apart thanks to the wing pattern and the presence of dark brown markings on the meso- and metathorax. *Spilosmylus tephrodestigma* sp. n. also lacks the amber shadings typical of *S. inquinatus* and *S. formosus*. The discovery of the genitalia of the new species is necessary to clarify its affinities within the genus.

### Key to the species of *Spilosmylus* known from the Philippines

**Table:** 

1	Forewing with embossed spot (Fig. 2B)	**2**
–	Forewing without embossed spot (Fig. 2A)	**8**
2	Forewing radial and medial area with dark grey spots in the medial area (Fig. 2B)	***S. tephrodestigma* sp. n.**
	Forewing radial and medial area without such markings	**3**
3	Forewing with diffuse amber shadings (Fig. 6B)	**4**
–	Forewing without amber shadings	**5**
4	Forewing veins Sc and R with 5 dark dashes, subcostal area unmarked	***S. inquinatus* (McLachlan)**
–	Forewing veins Sc and R with 2 dark dashes, subcostal area with two distinct, large dark brown markings covering the dark dashes; embossed spot very large (Fig. 6B)	***S. formosus* Banks**
5	Forewing subcostal area with several markings paralleling the dark dashes on Sc and R (Fig. 6A)	**6**
–	Forewing subcostal area mostly unmarked (Fig. 6D)	**7**
6	Forewing with an isolated dark spot in the medial area (Fig. 6C)	***S. apoanus* Banks**
–	Forewing without such a spot (Fig. 6A)	***S. alticolus* Banks**
7	Forewing veins Sc and R with 2 dark dashes; medial fork basal to the origin of the first branch of Rs (Fig. 6D)	***S. proximus* Banks**
–	Forewing veins Sc and R with 5 dark dashes; medial fork in proximity or slightly distal to the origin of the first branch of Rs	***S. modestus* (Gerstaecker)**
8	Forewing veins Sc and R with 7 dark dashes, subcostal area yellow and unmarked; forewing membrane with 3 large and well distinct light brown markings (Fig. 2A)	***S. spilopteryx* sp. n.**
–	Forewing subcostal area with dark streaks also covering Sc and R; forewing membrane shaded with dark brown along the outer gradates, rhegma and in proximity of the crossveins of the cubital and medial area but without distinct markings (Fig. 6E)	***S. monticolus* (Banks)**

Note: Navás (1926) described a further species of *Spilosmylus* from the Philippines: *S. nephelius* Navás, 1926. The holotype of this species, which was deposited in the private collection of the author, was likely destroyed (c.f. Monserrat 1985). Banks (1937) considered it a probable synonym of *S. inquinatus*.

**Figure 6.**
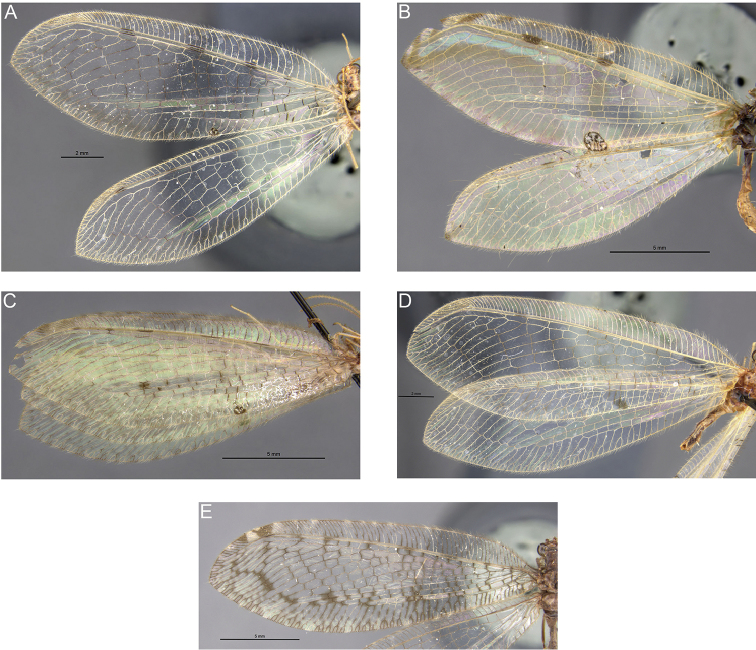
Detail of wings of the holotypes of *Spilosmylus* species described by Nathan Banks from the Philippines. Photographs by Philip D. Perkins, Museum of Comparative Zoology, Harvard University; original photography ^©^ President and Fellows of Harvard College. **A** *Spilosmylus alticolus* Banks, 1937 **B** *Spilosmylus formosus* Banks, 1924 **C** *Spilosmylus apoanus* Banks, 1937 **D** *Spilosmylus proximus* Banks, 1937 **E** *Spilosmylus monticolus* (Banks, 1937).

## Supplementary Material

XML Treatment for Spilosmylus spilopteryx

XML Treatment for Spilosmylus tephrodestigma
